# Flavonoid supplementation affects the expression of genes involved in cell wall formation and lignification metabolism and increases sugar content and saccharification in the fast-growing *eucalyptus* hybrid *E. urophylla x E. grandis*

**DOI:** 10.1186/s12870-014-0301-8

**Published:** 2014-11-19

**Authors:** Jorge Lepikson-Neto, Leandro C Nascimento, Marcela M Salazar, Eduardo LO Camargo, João PF Cairo, Paulo J Teixeira, Wesley L Marques, Fabio M Squina, Piotr Mieczkowski, Ana C Deckmann, Gonçalo AG Pereira

**Affiliations:** Departamento de Genética e Evolução, Laboratório de Genômica e Expressão, Instituto de Biologia, Universidade Estadual de Campinas, Campinas, São Paulo Brazil; Laboratório Nacional de Ciência e Tecnologia do Bioetanol, CTBE, Campinas, São Paulo Brazil; Department of Genetics, School of Medicine, University of North Carolina at Chapel Hill (UNC), Chapel Hill, NC USA

**Keywords:** Eucalyptus, Lignin, Phenylpropanoid metabolism, Syringyl/guaiacyl ratio, Gene expression, Hydrolysis, Stress

## Abstract

**Background:**

*Eucalyptus* species are the most widely planted hardwood species in the world and are renowned for their rapid growth and adaptability. In Brazil, one of the most widely grown *Eucalyptus* cultivars is the fast-growing *Eucalyptus urophylla x Eucalyptus grandis* hybrid*.* In a previous study, we described a chemical characterization of these hybrids when subjected to flavonoid supplementation on 2 distinct timetables, and our results revealed marked differences between the wood composition of the treated and untreated trees.

**Results:**

In this work, we report the transcriptional responses occurring in these trees that may be related to the observed chemical differences. Gene expression was analysed through mRNA-sequencing, and notably, compared to control trees, the treated trees display differential down-regulation of cell wall formation pathways such as phenylpropanoid metabolism as well as differential expression of genes involved in sucrose, starch and minor CHO metabolism and genes that play a role in several stress and environmental responses. We also performed enzymatic hydrolysis of wood samples from the different treatments, and the results indicated higher sugar contents and glucose yields in the flavonoid-treated plants.

**Conclusions:**

Our results further illustrate the potential use of flavonoids as a nutritional complement for modifying Eucalyptus wood, since, supplementation with flavonoids alters its chemical composition, gene expression and increases saccharification probably as part of a stress response.

**Electronic supplementary material:**

The online version of this article (doi:10.1186/s12870-014-0301-8) contains supplementary material, which is available to authorized users.

## Background

Trees constitute the majority of the lignocellulosic biomass on Earth and are expected to play a significant role in the future as a renewable and environmentally cost-effective alternative feedstock for biofuel production, a source of fibers and solid wood products and a major sink for excess atmospheric CO_2_ [1-3]. In Brazil, the pulp and paper industries have been efficiently fed by Eucalyptus forests due to their rapid growth, adaptability and wood quality, but with the dramatic increase in industrial demands and the interest in second-generation biofuels and renewable chemicals, the quality and quantity of wood produced must also increase [4,5].

Wood is a highly variable material that differs among trees and is composed of the secondary xylem, a specialized type of conductive and structural support tissue produced through the lateral growth and differentiation of the meristematic vascular cambium [6]. Most of the genes expressed during the formation of the secondary xylem (xylogenesis) are involved in determining the physical and chemical properties of wood [2,7]. Despite the progress that has been made in defining the molecular and cellular events involved in xylogenesis, the mechanisms regulating the rate of this process and the variation in wood properties remain largely unknown [8-10].

The secondary xylem cell wall of Eucalyptus trees is mostly composed by cellulose (β-1,4-glucan), lignin (a phenolic polymer) and hemicelluloses (heterogeneous polysaccharides), in an approximate ratio of 2:1:1 [11]. During tree growth, cellulose microfibrils give the cell walls tensile strength, and the lignin encasing the cellulose microfibrils imparts rigidity to the cell walls. Despite its importance during growth, lignin becomes problematic during postharvest, cellulose-based wood processing because it must be extracted during industrial handling through a complicated process, resulting in an enormous expenditure of energy and chemicals and strain on the environment [11,12]. Thus, it is of major interest to investigate the molecular basis of lignification to further increase our overall comprehension of this metabolic process for better adaptation of industrial processes.

Lignin synthesis is a relatively well-understood process that begins with the assembly of radicals produced during the single-electron oxidation of monolignols [10,13,14]. The industrial exploitation of wood to obtain cellulose depends mostly on the composition of lignins because lignins determine the rigidity of the wood and the feasibility of cellulose extraction, which are of major concern in the paper and pulp industries. In angiosperms, lignin is composed of 2 major units: the guaiacyl (G) and syringyl (S) units, which are derived from corresponding monolignol precursors, the coniferyl and sinapyl alcohols, respectively [1,15]. The S/G ratio dictates the degree and nature of polymeric cross-linking; an increased G content leads to highly cross-linked lignin (more rigid wood), whereas S subunits are typically linked through more labile ether bonds at the 4-hydroxyl position [16-18]. Thus, S-rich lignins are much easier to dissociate from cellulosic content, resulting in a much cleaner and cheaper process [18]. The S/G ratio is variable among species and is commonly used to evaluate the quality of wood in commercial tree plantations [19,20].

The formation of lignin monomers begins with the catalytic step performed by the 4-coumaroyl:CoA-ligase (4CL) enzyme, which likely represents the most important branch point in the central phenylpropanoid biosynthesis pathway in plants [21,22]. Through 4CL activity, cells can produce the precursors for either flavonoids or the G and S lignin precursors [23]. The product of 4CL, p-coumaroyl-CoA, is the substrate of the enzyme chalcone synthase (CHS) [24], which carries out the committing step in flavonoid biosynthesis. This pathway is reviewed in detail elsewhere [10,24].

The flavonoids naringenin-chalcone and naringenin, which are synthesized by the enzymes chalcone synthase (CHS) and chalcone isomerase (CHI), respectively, are the primary C15 intermediates in flavonoid biosynthesis [25,26]. This metabolic pathway appears to be a promising target for improving wood quality in Eucalyptus trees, as shown by our previous work [27] demonstrating that flavonoid supplementation of the fast-growing *Eucalyptus urophylla x Eucalyptus grandis* hybrid, hereafter referred to as *E. urograndis*, changes its wood composition, reduces its extractive contents and alters its syringyl monomer composition.

In this context, the objective of the present work was to perform further studies on the effects of flavonoid supplementation on *E. urograndis* trees by analyzing gene expression in xylem tissue from treated and non-treated trees and by measuring the effect on sugar accessibility through enzymatic hydrolysis. We analyzed the obtained data with special emphasis on results that might be correlated with the previously observed changes in wood composition [27].

## Results

### RNA sequencing and differential gene expression

A total of over 335 million reads were generated from 8 samples: 3 samples from the control group (CT); 3 from the naringenin-supplemented groups (2 NAR and 1 NARSTOP); and 2 from the naringenin-chalcone supplemented groups (1 CH and 1 CHSTOP). The number of reads per sample ranged from 32 to 54 million (total) and 30 to 48 million (after filtering). The reads were mapped against the greater splice variants (44,974 sequences) of the *E.grandis* gene predictions from Phytozome 7.0 (54,935 transcripts) using the SOAP2 alignment software package [28] (Additional file 1).

**Figure 1:**
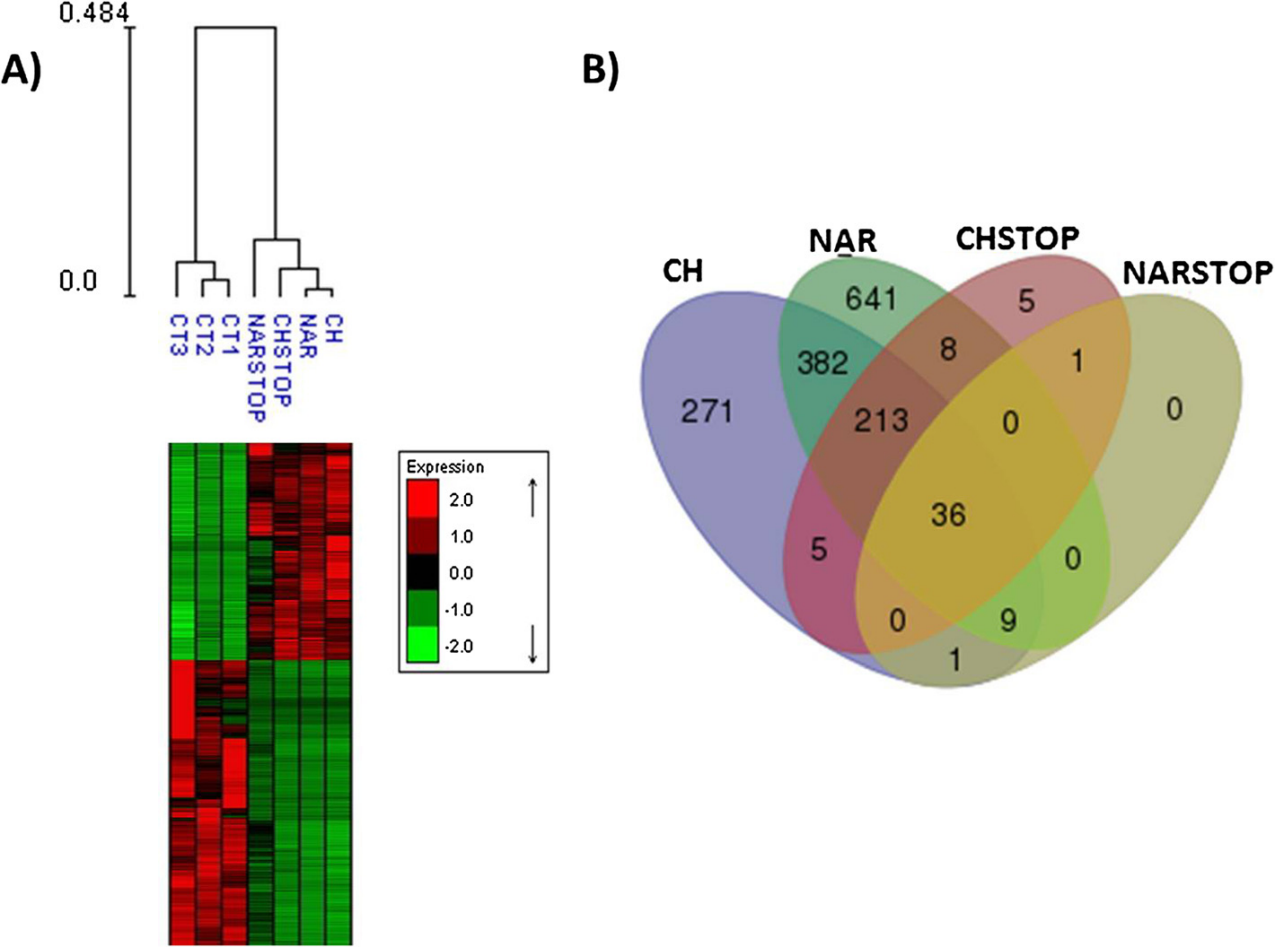
**Heat map clustering and Venn diagram of differentially expressed genes. A)** Heat map clustering of differentially expressed transcripts and comparison of the estimated log2 fold change correlations between each group subjected to differential expression analyses. **B)** Venn diagram of differentially expressed genes. CH- prolonged narigenin-chalcone supp; NAR – prolonged naringenin supp; CHSTOP- short-term naringenin-chalcone supp; NARSTOP – short-termnaringenin sup.

Heat map clustering of all transcripts was performed using Expander software [29], resulting in 2 major groups: 1 formed by the 3 control sample replicates and the other by the flavonoid-supplemented samples (Figure 1).

The read counts from each sample were used to test the differential expression of the genes between the control (CT) and supplemented (CH, NAR, CHSTOP and NARSTOP) treatments using the baySeq package [30]. A total of 1,573 genes were considered to be differentially expressed (FDR ≤0.01), which were distributed among the treatments (917 CH; 1,289 NAR; 268 CHSTOP; 47 NARSTOP) (Additional file 2).

The gene expression patterns observed for the supplemented and control groups were distinct, while similar profiles were observed within treatments, indicating similarities among the different types of flavonoid supplementation studied here. Most of the differences were observed in the long-term supplementation treatments, which comprised almost all of the genes that were differentially expressed in the short-term treatments as well. The NAR-supplemented plants displayed the greatest number of genes that were differentially expressed, while the NARSTOP-supplemented plants had fewer, which may indicate that naringenin supplementation has a stronger, but short-lasting impact on gene expression, whereas naringenin-chalcone has a smaller but more durable impact.

### Functional analyses

To determine the biological functions of the genes responding to flavonoid supplementation, functional analyses were performed using the web-based tools Blast2GO and Mapman. The genes considered differentially expressed in each treatment were mapped to their corresponding metabolic pathways, and the treatments were tested for enrichment of particular metabolic responses.

Only 36 genes were differentially expressed in all four treatments, including genes encoding several heat-shock proteins, sequences with no hits and unknown proteins (Table 1).

**Table 1:** **Gene ID, FPKM values and annotation of the 36 genes that found to be differentially expressed in all tested conditions**

		**FPKM**				
**Gene ID**	**Annotation**	**CT**	**CH**	**NAR**	**CHSTOP**	**NARSTOP**
Eucgr.F04479.1	HSP20	0.12	35.55	40.44	23.84	57.92
Eucgr.K02389.1	Unknown	0.04	13.69	10.18	9.89	26.99
Eucgr.K02399.1	Unknown	0.08	18.09	18.67	19.48	53.82
Eucgr.G01188.2	EGY3	2.78	45.78	41.56	33.40	76.35
Eucgr.J01979.1	HSP18.2	0.34	17.61	19.94	14.63	27.21
Eucgr.K02410.1	Unknown	0.13	14.27	10.66	12.52	29.61
Eucgr.J01980.1	HSP18.2	0.02	12.20	11.59	9.15	26.39
Eucgr.L02233.1	no hit	1.29	41.47	38.15	29.24	118.40
Eucgr.F02898.1	HSP20	2.76	525.99	343.17	280.68	342.59
Eucgr.J01985.1	HSP18.2	0.31	22.25	16.62	13.21	33.33
Eucgr.K03553.1	STS	0.04	4.11	4.59	2.83	6.87
Eucgr.L03261.1	HSP18.2	1.44	47.41	27.81	19,98	75,67
Eucgr.C03449.1	HSFA2	0.29	14.55	12.98	14.36	18.58
Eucgr.C00684.1	HSP17.6II	2.00	341.30	299.30	243.65	272.90
Eucgr.K02384.1	unknown	0.07	14.56	10.59	10.60	27.23
Eucgr.J01969.1	HSP20	4.89	192.23	134.53	103.09	310.44
Eucgr.K03472.1	ARATH	0.07	109.57	84.15	62.04	20.94
Eucgr.H04513.1	HSP70	0.23	15.72	18.79	11.56	21.62
Eucgr.A00595.1	PEBP	0.10	81.17	76.66	57.33	98.83
Eucgr.E02421.1	Unknown	0.19	260.04	220.12	119.63	51.05
Eucgr.H04692.1	HSP21	2.97	83.31	59.57	43.11	313.22
Eucgr.G02440.1	UGT73B2	0.00	5.46	5.80	3.33	4.06
Eucgr.G02259.1	UGT73B3	0.00	2.73	2.14	1.18	3.10
Eucgr.J01959.1	HSP18.2	3.19	142.90	89.76	58.22	148.21
Eucgr.K00295.1	HSP90.1	2.11	46.13	38.25	35.52	62.69
Eucgr.A01833.1	AAC3	0.13	32.00	24.43	15.08	10.47
Eucgr.C03071.1	HSP17.6II	3.64	517.08	509.51	451.66	324.54
Eucgr.B03843.1	No hit	1.45	93.17	63.39	67.61	20.77
Eucgr.C03320.1	DUF1677	0.38	24.39	18.22	14.01	9.78
Eucgr.B00176.2	PIMT2	3.86	153.25	109.01	82.97	57.39
Eucgr.J02588.1	No hit	3.20	225.90	182.76	194.01	130.04
Eucgr.C00690.1	HSP17.6II	2.48	563.68	498.40	514.67	458.94
Eucgr.K00237.1	PEBP	0.04	115.83	64.41	61.47	11.49
Eucgr.F03196.1	GSTU25	1.43	292.73	240.63	168.71	38.29
Eucgr.I02136.1	HSP20	1.68	226.73	147.43	90.02	259.35
Eucgr.H04427.1	MEE32	49.92	0.52	0.85	1.32	13.93

A total of 36 genes were differentially expressed in all four conditions. FPKM -fragments per kilobase of exon per million fragments mapped. CT – control; CH – prolonged naringenin-chalcone supp; NAR – prolonged naringenin supp; CHSTOP- short-term naringenin-chalcone supp; NARSTOP – short-term naringenin supp.

*Abbreviations*: *HSP20* HSP20-like chaperone superfamily protein, *unknown* unknown protein, *EGY3* ethylene-dependent gravitropism-deficient and yellow-green-like 3, *HSP18.2* heat shock protein 18.2, *HSP20* HSP20-like chaperones superfamily protein, *STS* stachyose synthase, *HSFA2* heat shock transcription factor A2, *HSP17.6II* 17.6 kDa class II heat shock protein, *ARATH* Adenine nucleotide alpha hydrolases-like superfamily protein, *HSP70* BIP1heat shock protein 70 family protein, *PEBP* phosphatidylethanolamine-binding protein family protein, *HSP21* heat shock protein 21, *UGT73B2* UDP-glucosyltransferase 73B2, *UGT73B3UDP* glucosyl transferase 73B3, *HSP90.1* heat shock protein 90.1, *AAC3* ADP/ATP carrier 3, *DUF1677* protein of unknown function, *PIMT2* protein-l-isoaspartate methyltransferase 2, *GSTU25* glutathione S-transferase TAU 25, MEE32 dehydroquinate dehydratase, putative/shikimate dehydrogenase.

**Figure 2:**
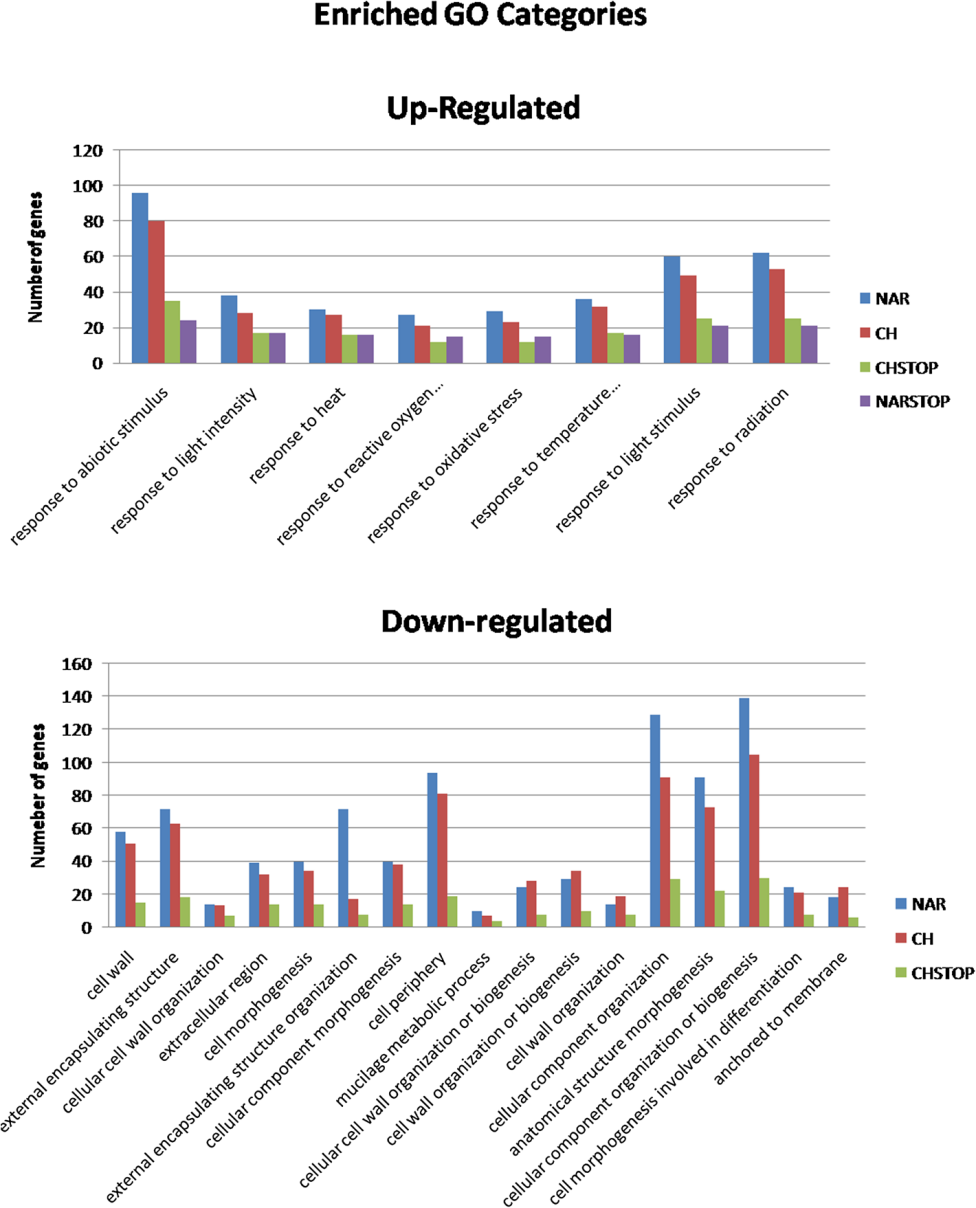
**GO analysis.** Common GO categories that were enriched (p-values ≤0.05) between treatments. CH – prolonged naringenin-chalcone supp; NAR – prolonged naringenin supp; CHSTOP- short term naringenin-chalcone supp; NARSTOP – short-term naringenin supplementation.

Each supplemented group was analysed individually. Common categories between different treatments are shown in Figure 2, and all affected GO categories are listed in Additional file 3.

Many of the down-regulated categories that were common to all treatments are involved in cell wall formation and development. On the other hand, the common up-regulated categories are all related to stress and environmental responses. Interestingly, NARSTOP, which resulted in fewer differentially expressed genes, only led to enriched GO categories among up-regulated genes.

**Figure 3:**
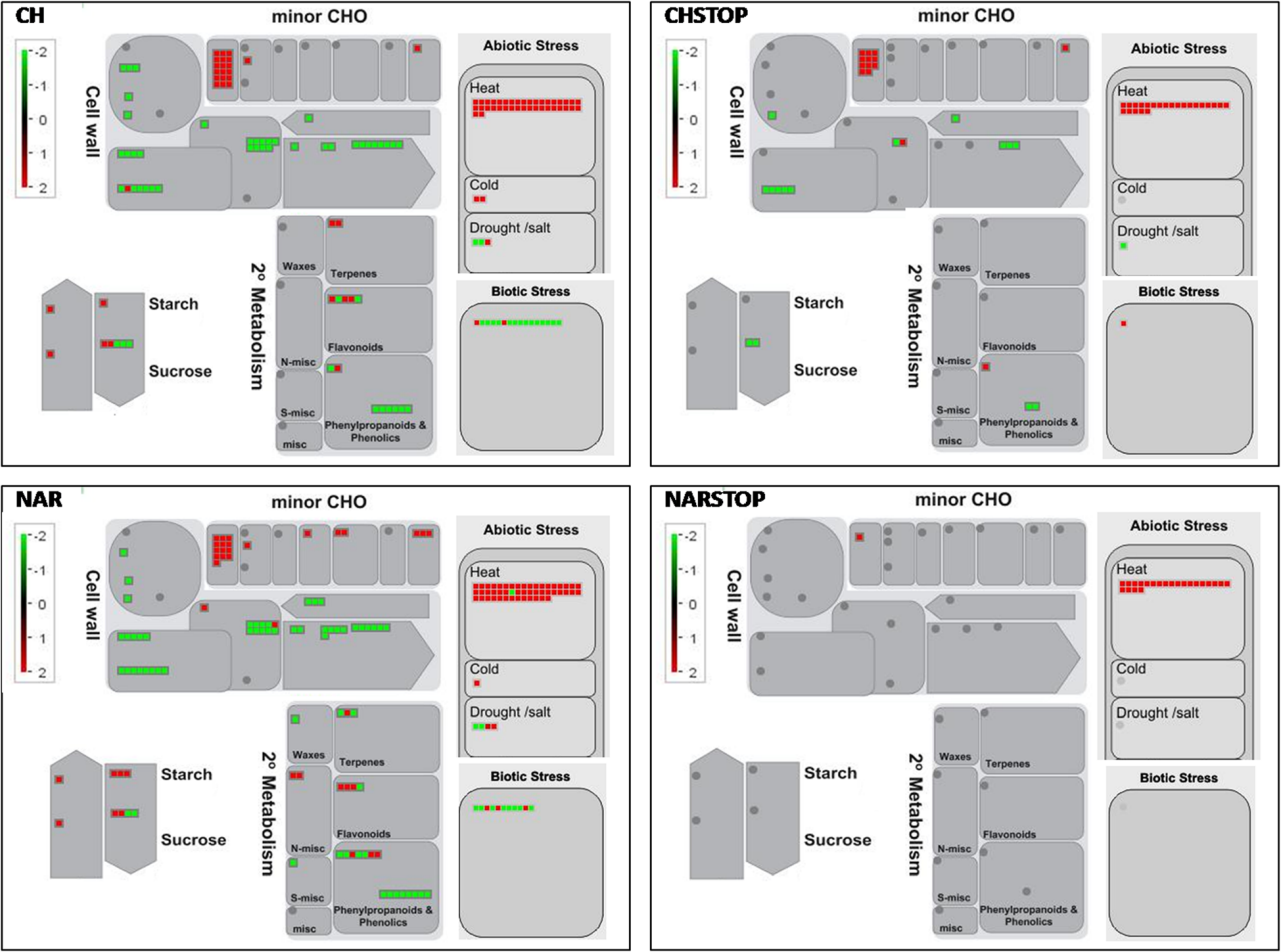
**MapMan analysis.** MapMan overview of the metabolism- and cellular response-related genes among the 1,573 genes that were differentially expressed under the four different flavonoid treatments. The presented values are the fold changes between the treatment and control groups. CH – prolonged naringenin-chalcone supp; NAR – prolonged naringenin supp; CHSTOP- short-term naringenin-chalcone supp; NARSTOP – short-term naringenin supp.

Mapman analyses of all of the differentially expressed genes also indicated down-regulation of cell wall-related genes and phenylpropanoid pathways, whereas flavonoid, minor CHO and starch and sucrose metabolism and stress response were associated with the most genes up-regulated (Figure 3).

### The phenylpropanoid genes

To further analyze the impact of flavonoid supplementation on lignification, a broader analysis was performed on the genes from the phenylpropanoid pathway, especially those related to lignin biosynthesis.

Several phenylpropanoid genes were differentially expressed between the treated samples and controls (Table 2), including the following genes that are directly related to lignin synthesis: 4CL, HCT, 2 OMT-methyltransferases, CCR and 2 CAD genes; 4CL, HCT and CCR were down-regulated, while the 2 methyltransferases and CAD genes were up-regulated. Additionally, several laccases were down-regulated among the treatments. These results are highly significant in terms of explaining the higher S/G ratio found in supplemented plants.

**Table 2:** **Differentially expressed phenylpropanoid-related genes**

		**FPKM**				
**Gene ID**	**Annotation**	**CT**	**CH**	**NAR**	**CHSTOP**	**NARSTOP**
Eucgr.C00859.1	U91A1	0.00	1.60*	0.83	0.28	0.00
Eucgr.K00903.1	AAT	0.38	3.91	4.61*	3.45	1.26
Eucgr.K00901.1	AAT	0.83	0.03*	0.98	1.18	1.51
Eucgr.E01250.1	PRR1	38.77	3.72*	4.68*	7.66	21.15
Eucgr.B03781.1	AA	24.04	0.04*	0.86	1.39	9.77
Eucgr.D02454.1	DFR	0.05	3.10*	2.73*	1.32	0.48
Eucgr.G02325.1	DFR	2.51	28.24*	20.16*	21.06	9.55
Eucgr.F04163.1	LAC14	6.22	0.15	0.32*	0.57	1.82
Eucgr.F02646.1	LAC14	1.99	0.00*	0.02*	0.06*	0.44
Eucgr.F04162.1	LAC14	1.95	0.09	0.04*	0.06	0.72
Eucgr.H04937.1	LAC14	13.67	0.02*	0.15*	0.21	4.66
Eucgr.F04160.1	LAC14	17.17	0.04*	0.25*	0.26*	3.08
Eucgr.F02674.1	LAC14	7.13	0.28	0.27*	0.42	2.90
Eucgr.H04936.1	LAC14	8.51	0.04*	0.03*	0.08	2.50
Eucgr.B02796.1	LAC4	12.04	0.28*	1.95	3.31	5.69
Eucgr.K00957.1	ATOMT1	1.36	17.54	17.19*	10.55	15.16
Eucgr.A01877.1	OMT-like	0.00	0.31	0.79	1.75*	0.22
Eucgr.J00363.1	HCT	88.68	3.76*	16.43	29.57	53.35
Eucgr.B00137.1	4CL	12.31	1.83	2.50*	4.21	6.24
Eucgr.E00270.1	CCR	30.29	3.21	3.29*	4.03	8.00
Eucgr.G01350.2	CAD5	23.73	146.29*	126.30*	144.55	62.96
Eucgr.E01110.2	CAD1	4.34	59.21	49.17*	51.45	27.12

FPKM -fragments per kilobase of exon per million fragments mapped. CT – control; CH – prolonged naringenin-chalcone supp; NAR – prolonged naringenin supp; CHSTOP- short-term naringenin-chalcone supp; NARSTOP – short-term naringenin sup *Denotes differential expression.

*Abbreviations*: *U91A1* UDP-Glycosyltransferase superfamily protein, *AAT* HXXXD-type acyl-transferase family protein, *PRR1* pinoresinol reductase, *AA* Plant L-ascorbate oxidase, *DFR* Dihydroflavonol-4-reductase, *LAC14* laccase 14, *LAC4* laccase 4, *ATOMT1* O-methyltransferase 1, *OMT-like* O-methyltransferase family protein, *HCT* hydroxycinnamoyl-CoA shikimate transferase, *4 CL* 4 coumarate CoA ligase, *CCR* cinnamoyl-CoA reductase, *CAD* cinnamyl alcohol dehydrogenase.

Interestingly, no gene related to the phenylpropanoid pathway was differentially expressed as a result of NARSTOP treatment.

### Secondary cell wall genes

In addition to genes from the phenylpropanoid pathway, many genes related to secondary cell wall formation were differentially expressed in response to flavonoid supplementation (Table 3). Among these genes, we observed sucrose synthases, cellulose synthases and many glucosylases and transferases, most of which were down-regulated following the prolonged supplementation treatments. However, we also observed the up-regulation of several genes related to secondary cell wall formation after both prolonged and short-term flavonoid supplementation, including galactinol synthase, stachyose synthase, raffinose synthase and starch synthase.

**Table 3:** **Differentially expressed secondary cell wall genes**

		**FPKM**				
**Gene ID**	**Annotation**	**CT**	**CH**	**NAR**	**CHSTOP**	**NARSTOP**
Eucgr.C03199.1	SUS4	1,532.75	66.91*	100.38*	132.04	213.15
Eucgr.C01715.1	SPS1F	3.32	63.28*	54.72	35.80*	28.00
Eucgr.F00464.1	SUT4	28.18	85.70	85.04	82.49*	36.89
Eucgr.D01765.2	CSLG3	0.07	3.26	6.28*	7.07*	1.24
Eucgr.F04010.1	CSLC05	7.51	0.11*	0.35	0.47*	3.06
Eucgr.J00420.1	CSLA2	41.08	2.63*	5.07*	5.87*	25.59
Eucgr.E00226.1	CSLD3	10.13	0.53	0.78	0.68	2.11
Eucgr.E00821.1	CSLG2	3.07	0.38*	0.35	0.78*	3.03
Eucgr.J02497.1	AMR1	1.00	5.25	6.14	6.11*	2.68
Eucgr.J02407.1	MUR1	74.28	18.89	17.82	19.95*	38.28
Eucgr.B03204.1	MUR2	13.62	55.51	54.92	47.91*	32.70
Eucgr.J01663.1	XTH5	97.41	1.21*	1.87*	3.31*	75.71
Eucgr.B03348.1	XTH33	26.89	0.21	0.15*	0.07*	9.47
Eucgr.K00883.2	XTH9	607.60	16.06*	29.74	37.61*	288.85
Eucgr.C00184.1	XTH23	45.26	0.45	0.72*	0.22*	37.20
Eucgr.H02634.1	XTH16	386.73	21.78*	47.14	72.69	396.09
Eucgr.D01294.1	XTH8	10.82	1.01	1.54	1.66*	7.36
Eucgr.J00827.1	GSL12	0.04	0.90*	1.56*	0.98*	0.76*
Eucgr.A02002.1	GSL7	0.20	1.24	2.13	1.38*	0.79
Eucgr.A02008.1	GSL7	0.16	1.07	1.89	1.66*	0.83
Eucgr.K02988.2	GH	16.20	90.51*	69.09*	53.94	47.27*
Eucgr.H00494.1	PWD	6.13	20.24	26.24*	27.22	7.21
Eucgr.H03767.1	BAM9	39.25	225.21	235.09*	217.12	115.07
Eucgr.E00460.1	TPS	0.08	5.75*	6.21*	4.26	0.34
Eucgr.K00387.1	SS	9.88	66.76*	54.46*	39.19	19.93
Eucgr.C04266.1	RafS	26.09	1,317.97*	1007.17	544.49	193.24
Eucgr.K03553.1	STS	0.04	4.11*	4.59*	2.83*	6.87*
Eucgr.H00997.1	STS	0.81	37.45*	29.83*	17.09*	7.06*
Eucgr.K03563.1	GoSL1	0.23	4.48	11.12*	12.00	8.48*
Eucgr.L00249.1	GoSL2	0.34	280.16*	135.36*	52.37*	1.25
Eucgr.L00243.1	GoSL2	0.02	29.83*	19.77*	9.75*	1.37*
Eucgr.L00251.1	GoSL2	0.21	325.56*	149.21*	61.46*	3.38
Eucgr.L03245.1	GoSL2	0.07	190.81*	124.71*	44.86*	2.79*
Eucgr.L00240.1	GoSL2	0.02	34.50*	22.77*	10.27*	0.66
Eucgr.L00248.1	GoSL2	0.17	162.07*	86.93*	32.28*	0.69
Eucgr.L03244.1	GoSL2	0.12	279.83*	137.40*	63.66*	2.99*
Eucgr.L00235.1	GoSL2	0.04	73.19*	38.88*	15.60*	0.03
Eucgr.L00245.1	GoSL2	1.83	287.87*	164.66	80.81*	5.28
Eucgr.F01661.1	Invertase	0.15	2.82*	2.16	2.39*	1.33
Eucgr.J00457.2	Invertase	5.69	47.20*	42.83	33.08*	18.78
Eucgr.G01751.1	Invertase	4.83	0.23*	0.42	1.17*	2.23
Eucgr.A02888.1	Invertase	7.36	0.04*	0.08*	0.08*	1.55

FPKM -fragments per kilobase of exon per million fragments mapped. CT – control; CH – prolonged naringenin-chalcone supp; NAR – prolonged naringenin supp; CHSTOP- short-term naringenin-chalcone supp; NARSTOP – short-term naringenin sup *Denotes differential expression.

*Abbreviations*: *Sus4* sucrose synthase 4, *SPS1F* sucrose phosphate synthase 1 F, *SUT4* sucrose transporter 4, *CSLG3* cellulose synthase like G3, *CSLD3* cellulose synthase-like D3, *CSLC05* Cellulose-synthase-like C5, *CSLA2* cellulose synthase-like A02, *CSLG2* cellulose synthase like G2, *CSLG3* cellulose synthase like G3, *CESA3* cellulose synthase family protein, *AMR1* ascorbic acid mannose pathway regulator 1, *MUR1* GDP-mannose 4,6 dehydratase 2, *MUR2* fucosyltransferase 1, *XTH5* xyloglucan endotransglucosylase/hydrolase 5, *XTH33* xyloglucosyl transferase 33, *XTH9* xyloglucan endotransglucosylase/hydrolase 9, *XTH23* xyloglucan endotransglycosylase 6, *XTH16* xyloglucan endotransglucosylase/hydrolase 16, *XTH8* xyloglucan endotransglucosylase/hydrolase 8, *GSL12* glucan synthase-like 12, *GSL7* glucan synthase-like 7, *GH* glycoside hydrolase, *PWD* phosphoglucan water dikinases, *BAM9* beta-amylase 9, *TPS* trehalose-6-phosphate synthase, *SS* starch synthase, *Rafs* raafinose synthase, *STS* stachyose synthase, *GoSL1* galactinol synthase 1, *GoSL2* galactinol synthase 2.

### Stress-related genes

Some of the most differentially expressed genes belonged to stress-related gene categories, which were up-regulated in all of the supplemented groups. These genes included several encoding heat-shock proteins and UDP-glycosil transferases (Table 4).

**Table 4:** **Differentially expressed stress-related genes**

		**FPKM**				
**Gene ID**	**Annotation**	**CT**	**CH**	**NAR**	**CHSTOP**	**NARSTOP**
Eucgr.H05081.4	ALDH3I1	4.84	0.93*	1.11*	1.60	5.34
Eucgr.C00112.1	CIA2	0.70	12.29*	14.05*	17.42*	3.57
Eucgr.K01387.2	COL9	3.33	26.78*	22.63*	18.02*	2.56
Eucgr.C03449.1	HSFA2	0.29	14.55*	12.98*	14.36*	18.58*
Eucgr.C03456.1	HSFA2	0.09	2.67*	3.64*	2.25*	1.45
Eucgr.C00873.1	HSFA2	1.92	24.38*	18.82*	14.75*	7.94
Eucgr.C03434.1	HSFA2	0.31	6.75*	6.22*	6.19*	2.03
Eucgr.C01043.1	HSFC1	2.92	122.26*	116.87*	96.98*	13.96
Eucgr.J01981.1	HSP18.2	2.00	34.43	50.34	69.86*	105.48*
Eucgr.J01980.1	HSP18.2	0.02	12.20*	11.59*	9.15*	26.39*
Eucgr.J01959.1	HSP18.2	3.19	142.90*	89.76*	58.22*	148.21*
Eucgr.J01958.1	HSP18.2	2.68	115.32*	90.34*	65.56	100.03
Eucgr.J01979.1	HSP18.2	0.34	17.61*	19.94*	14.63*	27.21*
Eucgr.J01958.1	HSP18.2	2.68	115.32*	90.34*	65.56	100.03
Eucgr.J01979.1	HSP18.2	0.34	17.61*	19.94*	14.63*	27.21*
Eucgr.J01977.1	HSP18.2	0.37	9.43*	8.59*	7.08	19.26*
Eucgr.J01964.1	HSP18.2	13.65	145.21*	119.05*	111.04	251.88*
Eucgr.J01985.1	HSP18.2	0.31	22.25*	16.62*	13.21*	33.33*
Eucgr.J01982.1	HSP18.2	0.21	8.65*	6.06*	4.24	13.03*
Eucgr.F04479.1	HSP20-like	0.12	35.55*	40.44*	23.84*	57.92*
Eucgr.I02136.1	HSP20-like	1.68	226.73*	147.43*	90.02*	259.35*
Eucgr.J01969.1	HSP20-like	4.89	192.23*	134.53*	103.09*	310.44*
Eucgr.G00061.1	HSP20-like	9.45	834.94*	858.21*	726.74*	814.87
Eucgr.E00433.1	HSP20-like	3.35	295.94	205.55*	185.03	165.89
Eucgr.F02898.1	HSP20-like	2.76	525.99*	343.17*	280.68*	342.59*
Eucgr.A01416.1	HSP21	0.08	12.40*	7.60*	4.34*	0.29
Eucgr.H04692.1	HSP21	2.97	83.31*	59.57*	43.11*	313.22*
Eucgr.J03127.1	Hsp70b	8.27	1576.75	1347.55*	881.92	925.42
Eucgr.H03518.1	HSP70T-2	6.06	282.04*	218.12*	152.91	249.47
Eucgr.K00295.1	HSP90-1	2.11	46.13*	38.25	35.52*	62.69*
Eucgr.F03704.1	MSL6	1.45	27.74	21.67*	11.53*	10.03
Eucgr.H02896.1	MYB305	0.07	6.57*	8.20*	6.34*	1.14
Eucgr.C00618.1	Oleosin	0.50	38.75*	24.66*	18.36*	2.85
Eucgr.F01003.1	PAT1	2.30	43.54*	47.40*	43.89*	6.02
Eucgr.K00237.1	PEBP	0.04	115.83*	64.41*	61.47*	11.49*
Eucgr.B00176.2	PIMT2	3.86	153.25*	109.01*	82.97*	57.39*
Eucgr.G01510.1	RLK	1.71	10.40*	11.17*	11.20*	6.80
Eucgr.F01854.1	TRX1	4.16	598.03*	295.93*	191.61*	25.01
Eucgr.G02440.1	UGT73B2	0.00	5.46*	5.80*	3.33*	4.06*
Eucgr.L03261.1	UGT73B3	1.44	47.41*	27.81*	19.98*	75.67*
Eucgr.G02259.1	UGT73B3	0.00	2.73*	2.14*	1.18*	3.10*
Eucgr.I00409.1	UGT73B3	0.06	3.27*	4.18*	2.91*	0.60
Eucgr.B02291.1	UGT76E11	10.86	52.79*	42.69*	39.45	45.69
Eucgr.K01389.2	XERICO	19.81	1,348.64*	796.35*	569.70*	120.14

FPKM -fragments per kilobase of exon per million fragments mapped. CT – control; CH – prolonged naringenin-chalcone supp; NAR – prolonged naringenin supp; CHSTOP- short-term naringenin-chalcone supp; NARSTOP – short-term naringenin sup *Denotes differential expression.

*Abbreviations*: *ALDH3I1* –aldehyde dehydrogenase 3I1, *CIA2* chloroplast import apparatus 2, *COL9* CONSTANS-like 9, *HSFA2* heat shock transcription factor A2, *HSFC1* heat shock transcription factor C1, *HSP18.2* heat shock protein 18.2, *HSP20* like chaperones superfamily protein, *HSP21* heat shock protein 21, *Hsp70b* heat shock protein 70B, *HSP70T-2* heat-shock protein 70 T-2, *HSP90.1* heat shock protein 90.1, *MSL6* mechanosensitive channel of small conductance-like 6, *MYB305* myb domain protein 305, Oleosin family protein; *PAT1* GRAS family transcription factor, *PEBP* –phosphatidylethanolamine-binding protein family protein, *RLK* receptor lectin kinase, *TRX1* thioredoxin H-type 1, *UGT73B2* UDP-glucosyltransferase 73B2, *UGT73B3* UDP-glucosyl transferase 73B3, *UGT76E11* UDP-glucosyl transferase 76E11, *XERICO* RING/U-box superfamily protein.

### Enzymatic hydrolysis

To verify the effects of flavonoid supplementation on sugar yields and saccharification in Eucalyptus wood, enzymatic hydrolysis was performed. The hydrolysates were analyzed for total sugar contents (‘reduced sugars’), which included most of the pentoses and hexoses from the hemicellulose fraction, and glucose content (‘glucose’), allowing an estimate of the percent of saccharification to be obtained.

Flavonoid-supplemented plantlets showed increased sugar and glucose values compared to the control groups. The reduced sugar content was increased from 50% (CH) to 250% (NARSTOP), and the glucose content was increased from 43% (CH) to 253% (NARSTOP). With the exception of the naringenin-chalcone prolonged supplementation treatment (CH), all of the treatment values were considered statistically significant (Table 5).

**Table 5:** **Total sugar and glucose values**

	**n**	**Reduced sugars (mg/ml)**	**Reduced sugar yield %**	**Glucose (mg/ml)**
CT	7	1.17 (0.67)	5.69 (3.23)	0.39 (0.23)
CH	4	1.8 (0.33)	9.06 (1.83)	0.56 (0.07)
NAR	3	2.54 (0.005)**	12.72 (0.66)**	0.87 (0.10)**
CHSTOP	3	2.32 (0.24)*	11.81 (1.08)*	0.85 (0.29)*
NARSTOP	3	3 (0.85)**	14.59 (4.34)**	0.99 (0.09)**

Mean values and standard deviations (parentheses) for total sugar and glucose levels. n –number of biological replicates; CT – control; CH – prolonged naringenin-chalcone supp; NAR – prolonged naringenin supp; CHSTOP –short-term naringenin-chalcone supp; NARSTOP – short-term naringenin supp. *p-value <0.05; **p-value <0.01.

## Discussion

The metabolism of phenylpropanoids follows 2 main pathways: the lignin branch and the flavonoid branch. The two pathways share common substrates and enzymes, and these shared components lead to a high level of interdependence between the pathways. Considering the economic interest in Eucalyptus trees for paper and pulp production, and given that flavonoids are known to have a direct influence on lignification and wood formation in several species [31,32], including Eucalyptus species, as previously demonstrated by our group [27], it is of high interest to verify the effects of flavonoid supplementation on gene expression, especially concerning genes related to wood formation. Additionally, there is a pressing interest in expanding the industrial uses of Eucalyptus because Eucalyptus forest cultures are well-established in Brazil and may affect other strategic sectors, such as second-generation biochemicals. In this case, Eucalyptus wood could be employed as lignocellulosic biomass for biological fermentation [33,34].

With this objective, we designed the present work to investigate the molecular basis of the differences in wood observed in flavonoid-supplemented *E. urograndis* trees. Additionally, in light of our previous findings, we paid special attention to the expression of genes involved with lignin and secondary cell wall formation and to the possible association between gene expression and the chemical composition of wood in Eucalyptus.

We analyzed the whole genome (44,974 genes) of Eucalyptus plants following supplementation with different flavonoids. A total of 1,573 (3,5%) differentially expressedgenes were identified, which were distributed among the supplementation groups: 963 genes were down-regulated and 610 genes were up-regulated. Most of the differentially expressed genes were associated with the prolonged supplementation groups (1,289 for NAR and 917 for CH), while the short-term supplementation groups displayed fewer differentially expressed genes (268 for CHSTOP and 47 for NARSTOP). Most of the differentially expressed genes in the CHSTOP and NARSTOP groups were also differentially expressed in the NAR and CH groups. Thus, naringenin supplementation appears to have had a stronger but less durable effect, while naringenin-chalcone supplementation has a longer-lasting effect on gene expression.

GO enrichment analyses demonstrated that there were several categories involved in cell wall formation that were down-regulated in all of the supplemented groups, including the phenylpropanoid pathway in the NAR-supplemented samples. The up-regulated gene categories included many responses to stress and the environment as well as genes related to sugar alcohols, through being involved in polyol, hexitol and alditol metabolism (minor CHOs), in the CH group. This pattern could also be observed in the mapping analysis of differentially expressed genes performed using MapMan software, in which several pathways, most notably those associated with the cell wall and phenylpropanoids, were down-regulated, while the metabolic pathways associated withminor CHOs, flavonoids, sucrose and starch displayed up-regulated genes. Furthermore, there was strong evidence that stress may play a major role, as several stress-related gene categories were found to be enriched via GO analysis, even in the groups subjected to short-term supplementation.

It was therefore clear that lignification and the phenylpropanoid pathway are affected by a great number of factors, and we believe that our work can help to clarify some of these factors. The interdependence of the phenylpropanoid, flavonoid and lignin branches has been explored in other studies. For example, it has been reported that 4CL activity is inhibited by some flavonoids, such as naringenin-chalcone and naringenin, which are the products of the *chalcone synthase* (CHS) and *chalcone isomerase* (CHI) enzymes, respectively [31,35]. The same study demonstrated that the administration of flavonoids suppressed the growth of 20 plant species, although the sensitivities of the plants to flavonoids were different.

In addition, the activation of the lignin precursor cinnamic acid (catalyzed by C4H) and p-coumaroyl-CoA (catalyzed by 4CL) is, to some extent, regulated by the activity of the CHS enzyme, which is involved in the first step of flavonoid biosynthesis [35]. It has also been reported that CHS is associated with growth suppression via the regulation of 4CL. This association has major importance in lignin biosynthesis in a great number of species [32,35].

As demonstrated by our results, several genes involved in the phenylpropanoid pathway were differentially expressed in plants subjected to supplementation with flavonoids (Table 2; Figure 3). Our most noteworthy findings revealed the differential expression of genes directly related to lignin synthesis. The NAR-supplemented group presented down-regulation of both the 4CL and CCR genes, whereas the ATOMT1 and 2 CAD genes were up-regulated. The CH-supplemented group exhibited HCT down-regulation and 1 CAD gene that was up-regulated. In the CHSTOP-supplemented group, 1 methyltransferase was up-regulated. No genes from the phenylpropanoid pathway were differentially expressed following supplementation with NARSTOP.

Surprisingly, the gene encoding F5H, which is one of the key enzymes involved in the synthesis of the monolignol sinapyl alcohol and, ultimately, the S lignin moiety, was not found to be differentially expressed on our analyses. This result is particularly interesting in light of our finding that the S/G ratios in all of the flavonoid-supplemented groups were higher than that of the control group. Thus, we expected a change in the expression of F5H following flavonoid treatment. Because phenylpropanoid metabolism is complex, it is likely that the differential regulation of other enzymatic steps, such as those encoded by the 4CL, HCT, CCR, ATOM1 and CAD genes, may underlie this response.

Some findings reported in the literature support this possibility. For example, 4CL plays a major role in phenylpropanoid metabolism, as its product, p-coumaroyl-CoA, is a substrate that is common to the flavonoid and lignin synthesis pathway. HCT silencing in Arabidopsis represses lignin synthesis and plant growth, and the metabolic flux is redirected toward flavonoids by chalcone synthase activity [24]. CCR catalyzes the reduction of hydroxycinnamoyl-CoA thioesters to the corresponding aldehydes; this reaction is considered to be a potential control point that regulates the overall carbon flux in favor of lignin [36]. Arabidopsis ATOMT1 knock-out mutants lack S units [37], and CAD catalyzes the reduction of cinnamaldehydes to cinnamyl alcohols, which is the last step in the biosynthesis of the monolignols, thus playing a pivotal role in determining the lignin monomer composition and increasing S contents [13].

There are also several laccases that have been demonstrated to be involved in lignification [38], and many laccases were found to be down-regulated in the NAR-, CH- and CHSTOP-supplemented samples.

Our results further corroborate those of [39], who suggested that Arabidopsis responds to the accumulation of 1 or more intermediates from the flavonoid pathway by down-regulating either the whole phenylpropanoid pathway or the specific branch leading to monocyclic phenolic compounds. According to our results, it is possible that the accumulation of naringenin-chalcone and naringenin, the products of CHS and CHI, respectively, due to exogenous supplementation, results in the down-regulation of genes from the phenylpropanoid pathway, with the exception of 2 genes involved in the final steps of sinapilic acid synthesis (ATOMT1 and CAD). This down-regulation may at least partially explain the higher S/G ratios observed in the supplemented samples and is in agreement with the findings of [40] that the reduction of total flux through the entire monolignol pathway affects G-lignin resulting in higher S/G ratio.

While the NARSTOP-supplemented plants did not show differential expression of any genes that are related to lignin synthesis according to our statistical analyses, they exhibited FPKM values that were similar to those of the NAR-, CH- and CHSTOP-supplemented groups, but closer to the control values than the other groups. This indicates that an early impact on gene expression may be sufficient to promote the phenotypic differences observed in this group.

Another possibility is that factors other than the genes from the lignification pathway per se influence the lignin monomer composition. Cook and collaborators [41] reported that the levels of cellulose, xylan and lignin are not completely dependent on the transcription of the genes involved in these metabolic pathways. Thus, the regulation of cell wall biosynthesis occurs at different levels, not only at the transcriptional level [41].

Additionally, other genes that have not yet been discovered may be causing the observed differences, as many no hits and unknown proteins were found among the most differentially expressed genes following flavonoid treatment. The stress and environmental response pathways were significantly enriched and associated with lignification; thus, these pathways may play major roles in the alterations of lignin composition after flavonoid supplementation.

Stress and lignification are closely related. Many of the products of the phenylpropanoid pathway are induced by biotic and abiotic stress [42]. Both flavonoids and sinapate esters, which are used for lignin synthesis, are important for UV protection [39]. Arabidopsis mutants with reduced levels of CHS and CHI activity show up to 60% higher levels of sinapate esters [39,42].

Moreover, a large number of phenylpropanoids are induced by stress, such as those derived from the C15 flavonoid skeleton that are synthesized via the chalcone synthase (CHS)-mediated condensation of p-coumaroyl-coenzyme A (CoA) and three molecules of malonyl-CoA [43]. In most plant families, the initial product of CHS is a tetrahydroxychalcone, which is further converted to other flavonoid classes, such as flavones, flavanones, flavanols, anthocyanins and 3-deoxyanthocyanidins, all of which are compounds that are important in the response to stress [44].

The observation that several genes related to stress responses are differentially expressed in flavonoid-supplemented trees confirms the importance of stress responses in defining Eucalyptus wood properties as has been previously shown by our group [45]. In this work, a comparison between three Eucalyptus species revealed differential expression of stress-related genes in *E. urophylla*, which could explain the higher plasticity and adaptability of this species compared to *E. grandis* and *E. globulus*, the two other studied species.

In the present study, the stress-related genes that were differentially expressed following flavonoid treatments included a noteworthy group composed of several UDP-glucosyltransferases (UGTs), which were up-regulated in all of our treatments. In plants, UGTs utilize UDP-glucose, UDP-galactose, and UDP-rhamnose as sugar donors and are involved in the modulation of plant architecture and the water stress response in Arabidopsis [46]. The glucosylation of coniferyl aldehyde and sinapyl aldehyde may regulate both lignin biosynthesis and the metabolism of other phenylpropanoids, such as ferulic acid, 5-hydroxyferulic acid, sinapic acid and their derivatives [47]. Thus, the presence of up-regulated UGTs in all of the groups is another interesting result that might help elucidate the chemical differences present in the flavonoid-supplemented trees.

Another notable finding regarding cell wall formation was the differential expression of genes involved in the metabolism of sucrose, starch, CHOs and minor sugars. Despite the down-regulation of sucrose synthase (Sus) and cellulose synthase (CesA), there were several other enzymes involved in this metabolic pathway that were up-regulated in the prolonged flavonoid treatments, even in the short-term treatments. Starting with galactinol synthase (GolS2), which was one of the most differentially expressed genes, all of the downstream genes in this pathway were differentially expressed (up-regulated), most of which were up-regulated after both the prolonged and short-term treatments.

**Figure 4:**
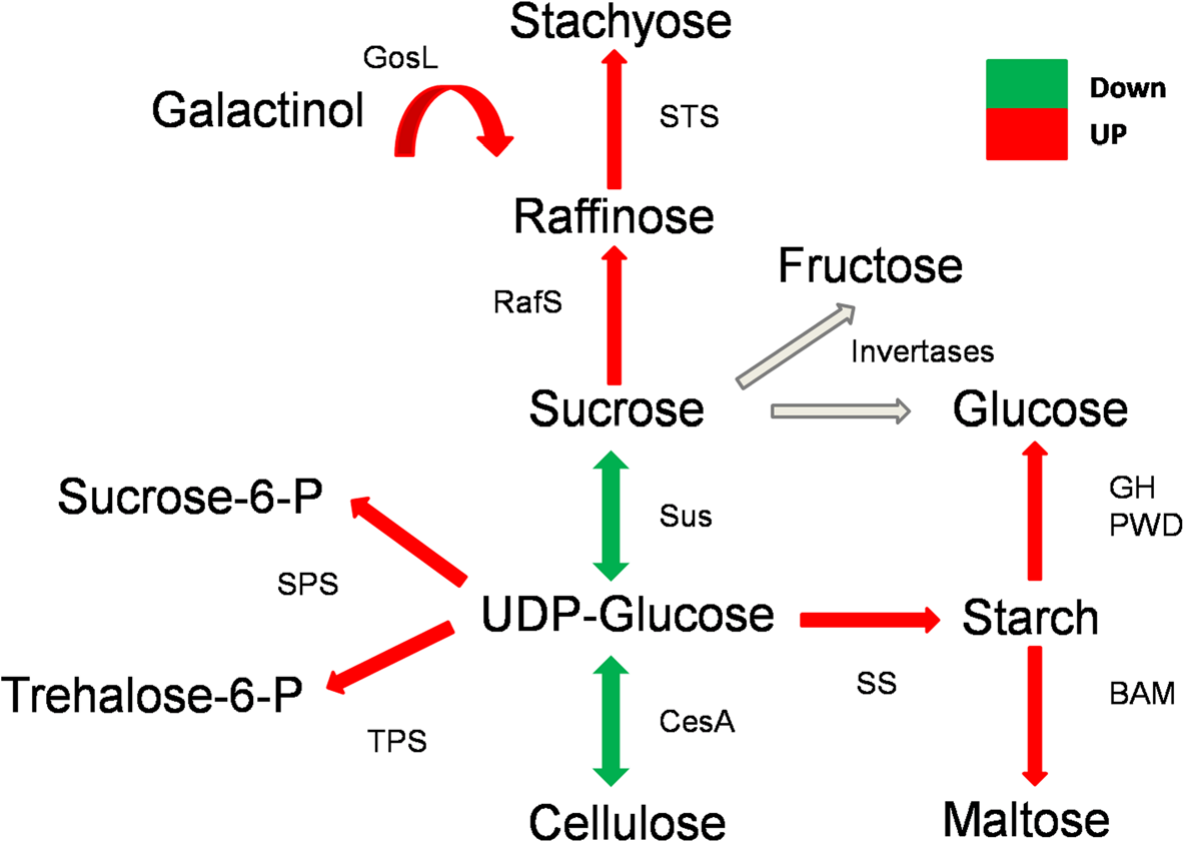
**Effects of flanonoid supplementantion on secondary cell wall related genes.** The effects of flavonoid supplementation on the expression of secondary cell wall-related genes. Sus –sucrose synthase; SPS –sucrose phosphate synthase; CesA –cellulose synthase;; GH –glycoside hydrolase; PWD –phosphoglucan water dikinases; BAM –beta-amylase; TPS –trehalose-6-phosphate synthase; SS –starch synthase; Rafs – raafinose synthase; STS –stachyose synthase; GoSL –galactinol synthase.

The most notable of these genes was stachyose synthase (STS), which converts raafinose (a trisaccharide of galactose, fructose and glucose) into stachyose (a tetrasaccharide) by transferring a galactosyl moiety from galactinol, [48]. Raafinose synthase (RafS) was also differentially up-regulated. Additionally, sucrose phosphate synthase (SPS1F), which catalyses the conversion of UDP-glucose and D-fructose 6-phosphate into UDP and sucrose 6-phosphate [49], was also differentially up-regulated (Figure 4).

Starch synthase (SS) was up-regulated as well, as were enzymes involved in the degradation of starch into maltose (beta-amylase; BAM) and glucose (glycoside hydrolase; GH and phosphoglucan water dikinases; PWD). These results suggest a shift from sucrose and cellulose production to the synthesis of starch and minor sugars (galactinol, raffinose and stachyose).

Thus, to verify the possible effects of these transcriptional responses on cell wall formation and sugar accessibility, enzymatic hydrolysis was performed in wood samples from all of the experimental and control plants. The results revealed an increase in sugar contents (up to 250% in the NARSTOP group) and glucose yields of all of the flavonoid-supplemented Eucalyptus plants.

This may be the result of the plants producing more sugar or a result of the increased digestibility of lignin due to modifications of lignin structure and other cell wall components. The total values should increase exponentially with the use of pre-treatments and the extension of milling times [50,51].

Galactinol, raffinose and stachyose have been described as being involved in freezing and stress tolerance [52,53]. The levels of galactinol and raffinose are increased in the leaves of Arabidops is plants over-expressing HsfA2, a heat shock factor, suggesting a possible role for these compounds in protection from oxidative damage [54]. Moreover, this factor may also constitute another link between the stress response and cell wall formation in flavonoid-supplemented Eucalyptus plants because all of our treatment groups showed more than 1 up-regulated HsfA2 gene.

Our results are also in agreement with those of [55], who verified that in poplar trees, the over-expression of GolS and its product, galactinol, may serve as a molecular signal that initiates metabolic changes associated with combating stress, culminating in the formation of tension wood and increased glucose contents. Additionally, over-expression of raffinose synthase was found to result in increased biomass and total cellulose contents, while the total contents of lignin and xylose moieties were slightly reduced. Furthermore, the total amount of glucose was commensurately increased in the transgenic trees, by from ~1 to 4% [55]. Moreover, repression of the flavonoid pathway in Arabidopsis increases starch levels [56], and a chalcone isomerase-deficient Arabidopsis mutant exhibits increased levels of starch and soluble sugars in its leaves.

Based on our results, flavonoid supplementation causes a stress response in *E. urograndis*, greatly affecting cell wall development, modifying lignification by affecting the expression of genes involved in the phenylpropanoid pathway and altering sugar metabolismin favor of starch and minor sugar synthesis, resulting in increased sugar accessibility and saccharification.

## Conclusions

The effects of flavonoid supplementation on cell wall development in Eucalyptus plants are most likely due to a combination of transcriptional changes in several distinct pathways. The down-regulation of the phenylpropanoid pathway, combined with the up-regulation of ATOMT1 and CAD, results in a higher S/G ratio, which in turn, increases lignin solubility and facilitates access to cellulose and hemicellulose. Subsequently, as a result of the stress response, sugar metabolism is shifted towards starch and minor sugars, culminating in the increased sugar and saccharification levels identified due to hydrolysis.

Given the importance of Eucalyptus in several industrial sectors, there is great interest in expanding the use of these species as a resource for cellulose, paper and pulp production and as an alternative source of biomass for second-generation biochemicals. Our results contribute not only to our understanding of the molecular responses involved in wood formation but will also have a significant impact on the use of Eucalyptus as biomass. Finally, we expect our findings to guide future genetic manipulation and nutritional supplementation analyses of Eucalyptus trees aimed at achieving significant improvements in their productivity yields.

## Methods

### Plant materials and tissue harvesting

Plantlets of a 6-month-old commercial clone of *Eucalyptus urograndis* were provided by International Paper (Mogi-Guaçu, Brazil) and grown in a greenhouse. The plantlets were divided into 5 groups according to supplementation conditions (apart from the standard nutritional solution supplied to all groups), as follows: control group (CT); experimental group 1 (CH), supplemented with 0.1 mmol of naringenin-chalcone for 5 months; experimental group 2 (NAR), supplemented with 0.1 mmol of naringenin for 5 months; experimental group 3 (CHSTOP), supplemented with 0.1 mmol of naringenin-chalcone for only the first month and then given the standard nutrition solution for the next 5 months; and experimental group 4 (NARSTOP), supplemented with 0.1 mmol of naringenin for only the first month and then given the standard nutrition solution for the next 5 months. Approximately 100–150 mL of each solution was administered via root application daily. The treatments lasted 5 months. The composition of the standard nutritional solution has been described previously [57]. At the end of the experiment, all 5 groups of plantlets were cut, and their stems were debarked, immediately frozen in liquid nitrogen and kept at −80°C for analysis; no growth differences were observed between the control and treatment groups (Additional file 4). All samples were analyzed 5 months after the beginning of the experiment, regardless of the applied supplementation.

Naringenin (4′-,5-,7-trihydroxyflavanone, 95%) and naringenin-chalcone (1,3-diphenyl-2-propen-1-one, 97%) were purchased from Sigma-Aldrich Co. (Tokyo, Japan) and AcrosOrganics Co. (Tokyo, Japan), respectively.

### Total RNA extraction

Total RNA was extracted according to the protocol described by [58], with the modifications proposed by [59]. The obtained RNA concentration and quality were verified using a Nanodrop 2000 spectrophotometer (Thermo Scientific).

### mRNA sequencing

mRNA sequencing was performed at the High-Throughput Sequencing Facility of the Carolina Center for Genome Sciences (University of North Carolina, USA). From each xylem sample, 10 μg of total RNA was used to prepare an mRNAseq library according to the protocol provided by Illumina. The gel extraction step was modified by dissolving excised gel slices at room temperature to avoid underrepresentation of AT-rich sequences [60]. Quality control and quantification of the libraries were performed using a DNA 1000 series II Bioanalyzer Chip (Agilent). For each library, single-end sequences of 36 or 50 bp were generated in a single lane using an Illumina Genome Analyzer IIx. A total of 8 libraries were generated: 3 biological replicates of the control group (CT); 2 biological replicates of the 5-month naringenin-supplemented groups (NAR); and 1 library for each remaining group (subjected to 1 month of supplementation with naringenin (NARSTOP), 5 months of supplementation with naringenin-chalcone (CH) or 1 month of supplementation with naringenin-chalcone (CHSTOP). Each library was constructed from a sample pooled from 3 individual trees. The complete dataset of RNA-seq reads has been deposited in SRA under accession numbers SRS716289; SRS716288, SRS716285; SRS716286; SRS716284.

### Read alignment

The obtained Illumina reads were filtered to exclude ribosomal sequences (using the SILVA database [61] and low quality reads (phred ≥20). The remaining reads were aligned against the greater splice variants of *E. grandis* transcripts from Phytozome 7.0 (44,974 sequences) available at (http://www.phytozome.net/) [62] using the SOAP2 alignment software package [28]. To prepare the data for Genebrowser analysis, the read were aligned to the *E. grandis* genome using the TopHat aligner [63] to allow for spliced alignments. Both programs were configured to allow up two mismatches (because SNPs can generate mismatches in the alignments, especially in cases such as the present analysis, where the sequences come from different species), discard sequences with ambiguities (Ns) and return only reads with unique alignments.

### Gene annotation

The Autofact program [64] was used to perform an automatic annotation of all *E. grandis* transcripts. The main feature of Autofact is its ability to perform gene annotation based on sequence similarity searches of several databases. BLASTx [65] (e-value cutoff of 1e-5) was used to align the obtained contigs against the following public databases: the NCBI non-redundant (NR) database; the Uniref90 and Uniref100 databases, which contain clustered sets of proteins from Uniprot [66]; the KEGG database of metabolic pathways [67]; and TAIR (version 10), an Arabidopsis proteins database. Functional annotation (GO) was performed using BLAST2GO [68] and MaPMAN [69] with the default parameters.

### Determination of gene expression levels

Gene expression was measured via the FPKM (fragments per kilobase of exon per million fragments mapped) method using only one read alignment for each transcript, as described by [70]. The FPKM values for all transcripts are available for searching in the EUCANEXT database (www.lge.ibi.unicamp.br/eucalyptusdb).

### Enzymatic hydrolysis

#### Substrate preparation

Samples from each Eucalyptus treatment were frozen in liquid nitrogen and then dried in FreeZone6 (Labconco) at - 51°C and 25 Pa for 48 hours. Subsequently, the lignocellulosic material was reduced through 1 cycle of 5 minutes in a ball-milling reactor. The milled material was used as a substrate for fungal growth and hydrolysis assays.

### Secretome production for enzymatic hydrolysis

The *Neurospora crassa* wide strain St.L. 74A (Missouri University, Kansas City, http://www.fgsc.net/) was used for secretome production. Conidia preparation was performed by inoculating the fungus in 100 mL of minimal medium plus Vogel’s salts supplemented with 143 μL of biotin (biotin 5 mg, ethanol 50% (v/v), 143 μL of a trace element solution (5 g monohydrate citric acid, 5 g of ZnSO4.7H2O, 1 g of Fe (NH4)2.6H2O, 0.25 g of CuSO4.5H2O, 0.05 g MnSO4.H2O, 0.05 g H3BO3, 0.05 g Na2MoO4.2H2O, qsp 1,000 mL), 1.5% agar and 2% sucrose. The prepared samples were grown for 3 days at 30°C in the dark and then for 7 days in the light at 25°C. A conidial suspension was then inoculated in 100 mL of the same medium described above without agar [71] containing as the only carbon source 2% of a substrate blend of 3 Eucalyptus species: *E. grandis, E. urograndis and E. urophylla*, in a ratio of 3:3:1, prepared as described above.

### Eucalyptus hydrolysis

Hydrolysis was performed as described by [72] with the following modifications: enzymatic hydrolysis was performed in 2 mL tubes shaken at 1,000 rpm at 30°C in a Thermomixer (Eppendorf) for 48 hours. Approximately 10 mg of substrate from each substrate preparation was diluted in 400 μL of 50 mM sodium acetate buffer pH 5.5, and 100 μL of the *N. crassa* secretome was then added. The protein concentration of the secretome was 0.4 μg/μL, as determined in a Bradford Kit assay (BioRad) with BSA as a standard. This temperature and pH were optimal for the hydrolysis of carboxymethyl cellulose, xylan and ß-glucan, as determined by testing the temperatures of 25–40°C and pH levels of 4.0 − 9.0. All hydrolysis reactions were performed in triplicate. For determination of the reducing sugar concentration and glucose production in supernatants derived from Eucalyptus hydrolysis, 2 mL tubes were centrifuged at 20,000 × g for 10 minutes at 4°C. The supernatant was then recovered, and 100 μL of each reaction was used to determine the content of reducing sugars by adding 100 μL of the dinitrosalicylic acid (DNS) assay reagent [73] heated to 99°C. A 100 μl aliquot of the sample was next transferred to an ELISA plate, and its absorbance was measured at 540 nm using a Tecan Infinite M200 microplate reader, referring to calibration curves generated from glucose solutions. To calculate glucose contents, 20 μL of the supernatant was added to 100 μL of a working solution from a Glucose Oxidase Kit (Laborlab) in ELISA plates. The reaction was subsequently incubated at 37°C for 10 minutes, and its absorbance and measured at 505 nm (in a Tecan Infinite M200 microplate reader). Glucose concentrations were calculated with a factor referring to a standard solution of glucose at 1 mg/mL. A blank reaction containing only buffer and substrate was subtracted from the measurements obtained for each assay. To verify significant differences between the controls and the flavonoid-supplemented groups, a one way ANOVA test was performed between the control and each supplemented group. The results were considered significant if p < 0.05 and were classified as follows: *, p < 0.05; **, p < 0.01.

### Supporting data

The data set(s) supporting the results of this article is (are) included within the article (and its additional file (s)). The complete dataset of RNA-seq reads has been deposited in SRA under accession numbers: SRS716289; SRS716288, SRS716285; SRS716286; SRS716284. The FPKM values for all transcripts are available for searching in the EUCANEXT database (www.lge.ibi.unicamp.br/eucalyptusdb).

## Additional files

Additional file 1:
**All expressed genes on any condition.**
Additional file 2:
**All differential expressed genes on all conditions.**
Additional file 3:
**Complete gene onthology analysis.**
Additional file 4:
**Height and diameter of samples.**
