# Supplementary material for: Flavonoid supplementation affects the expression of genes involved in cell wall formation and lignification metabolism and increases sugar content and saccharification in the fast-growing eucalyptus hybrid E. urophylla x E. grandis
Source: BMC Plant Biol. 2014 Nov 19;14:301. doi: 10.1186/s12870-014-0301-8 (PMC4248463; doi:10.1186/s12870-014-0301-8)
Supplement: Additional file 4: — Height and diameter of samples. [file 12870_2014_301_MOESM4_ESM.docx]

## Table 6 - Measurements of height and diameter

Mean values and standard deviations (parentheses) for height and diameter; CT – control; CH – prolonged naringenin-chalcone supp; NAR – prolonged naringenin supp; CHSTOP –short-term naringenin-chalcone supp; NARSTOP – short-term naringenin supp.

|  |  | CT | CH | NAR | NARSTOP | CHSTOP |
| --- | --- | --- | --- | --- | --- | --- |
| Height 3 months(m) | | 0,44(0,03) | 0,49(0,03) | 0,47(0,01) | 0,47(0,03) | 0,47(0,04) |
| Height 6 months(m) | | 1,32(0,02) | 1,28(0,02) | 1,30(0,04) | 1,35(0,03) | 1,29(0,03) |
| Diameter 1 month(mm) | | 6,05(0,04) | 6,00(0,04) | 5,92(0,11) | NA | NA |
